# Measurement properties of the Swedish version of the Knee Self‐Efficacy Scale: A Rasch analysis

**DOI:** 10.1002/jeo2.70306

**Published:** 2025-06-11

**Authors:** Ramana Piussi, Roland Thomeé, Kristian Samuelsson, Eric Hamrin Senorski

**Affiliations:** ^1^ Unit of Physiotherapy, Department of Health and Rehabilitation, Institute of Neuroscience and Physiology, Sahlgrenska Academy University of Gothenburg Gothenburg Sweden; ^2^ Sahlgrenska Sports Medicine Center Sahlgrenska Academy Gothenburg Sweden; ^3^ Sportrehab Sports Medicine Clinic Gothenburg Sweden; ^4^ Department of Orthopaedics, Institute of Clinical Sciences, The Sahlgrenska Academy University of Gothenburg Gothenburg Sweden; ^5^ Swedish Olympic Committee Stockholm Sweden

**Keywords:** patient‐reported outcomes, psychometric properties, rasch analysis, rehabilitation outcomes

## Abstract

**Purpose:**

This study aimed to evaluate measurement properties of the Swedish version of the Knee Self‐Efficacy Scale 18 items version (K‐SES_18_) in patients after anterior cruciate ligament (ACL) reconstruction using Rasch measurement theory (RMT).

**Method:**

Data were extracted from Project ACL, a rehabilitation registry. Responses to the K‐SES_18_ from 1307 patients (aged 15–50 years) at 12 months post‐ACL reconstruction were analyzed. The RMT analysis assessed targeting aspects of reliability, response category thresholds, item hierarchy, overall and individual item fit, local dependency, differential item functioning (DIF) and unidimensionality.

**Results:**

The use of RMT indicated that the K‐SES_18_ present subscale exhibited high reliability (Person separation index = 0.904; Cronbach's *α* = 0.939) but also a potential ceiling effect (mean person location = 1.440). Disordered thresholds were observed in 11 out of 14 items, which indicates that, according to the RMT, patients might have some difficulties to distinguish between response categories. Item misfit was noted, which suggests multidimensionality. Subgroup analyses partially resolved local dependency but indicated distinct dimensions for daily activities, leisure activities and physical activities. The K‐SES_18_ future subscale also demonstrated good reliability (PSI = 0.750; *α* = 0.783) but showed targeting issues and multidimensionality across items.

**Conclusion:**

The main finding of this study is that the K‐SES_18_, according to RMT, demonstrates strong reliability but has limitations in terms of measurement properties that possibly limit its clinical utility. The present and future subscales exhibit ceiling effects, disordered thresholds, and item misfit. Therefore, the K‐SES_18_ might not be able to capture the full range of patients' knee self‐efficacy after ACL reconstruction. These findings indicate that a scale refinement to enhance targeting, response sensitivity, and dimensionality is needed. Clinicians are therefore advised to interpret the subscale scores cautiously, acknowledging the multidimensional nature of the constructs measured.

**Level of Evidence:**

Level III.

**Abbreviations**
ACL: anterior cruciate ligament
CV: critical value
DIF: differential item functioning
K‐SES: knee self‐efficacy scale
PCA: principal component analysis
PRO: patient‐reported outcome
PSI: person separation index
RMT: rasch measurement theory
SD: standard deviation

## INTRODUCTION

After an injury and its treatment, the evaluation is a mandatory step in evidence‐based medicine [31]. In the field of anterior cruciate ligament (ACL) injury, recent guidelines stress the need for evaluation of patients with psychological patient‐reported outcomes (PROs) [2, 34]. One such PRO is the Knee Self‐efficacy Scale (K‐SES) for patients who suffer an ACL injury. The K‐SES aims to measure a patient's knee‐related self‐efficacy, defined as the belief in one's ability to perform a tasks [35]. The K‐SES was initially developed in 2006 and found to have good reliability, face validity and construct validity based on exploratory analyses [35]. However, a systematic review conducted in 2018 identified gaps in psychometric evidence for the K‐SES, including limited evidence for content validity and reliability due to methodological shortcomings [15]. The K‐SES showed unknown evidence for reliability, structural validity, and hypothesis testing, largely due to inadequate sample sizes and poor reporting in prior studies [15]. In 2021, an 18‐item version of the K‐SES was developed (K‐SES_18_), which showed similar or better properties compared with the original version [8].

An editorial published in 2022 calls researchers to take responsibility for using PROs of insufficient psychometric properties to inform decision making or to evaluate treatment outcomes [23]. Evidence for the psychometric properties of a PRO is built through time and through different trials. Within psychometric assessment, the tests used to determine a certain property can be divided into modern test theory and classic test theory [37]. While classic test theory uses Cronbach alpha, principal component analysis (PCA) or multitrait scaling, Rasch measurement theory (RMT) is an example of modern test theory, and is considered superior, since it provides a more sophisticated framework to assess measurement properties, and therefore allows for more precise evaluations [20, 33]. The RMT [30] is a mathematical model that translates ordinal observations to interval measurements and thereby generates quantitative values. Compared with correlation‐based models used in classic test theory, RMT is based on probability, that is, the probability for a certain individual to attain a certain value on an outcome measure. The use of an RMT analysis for the assessment of PROs in patients after ACL injury is an important piece in presenting evidence of PROs' psychometric properties. An example is the work of Comins et al. [13] on the Knee injury and Osteoarthritis Outcome Score (KOOS). However, RMT analyses on other commonly used PROs, such as the K‐SES_18_, are still lacking.

Therefore, the aim of this study was to analyze measurement properties, according to RMT, of the Swedish version of the K‐SES_18_ in patients after ACL reconstruction.

## METHODS

This study was reported according to the Rasch Reporting Guideline for Rehabilitation Research statement [26]. Data for the present study were prospectively collected in a rehabilitation outcome registry, Project ACL [19]. The registry was established in 2014 and aims to improve the care of patients who suffer an ACL injury. Data in Project ACL consists of results from muscle function tests and PROs collected prospectively before surgery (in case of ACL reconstruction), and thereafter at 10 weeks, 4, 8, 12, 18 and 24 months, and every 5 years with ACL injury/reconstruction as a baseline [7, 19]. Prior to participation in Project ACL, written consent was collected. Ethical approval was obtained from the Regional Ethical Review Board in Gothenburg (265‐13, T023‐17) and the Swedish Ethical Review Authority (2020‐02501).

### The Knee Self‐Efficacy Scale

The K‐SES was developed in 2006. Items were generated through clinical discussions between 12 physical therapists, 2 orthopaedic surgeons and 2 medical doctors. Some items were reported to be generated by ‘discussion between health care professionals and patients' [35]. After two pilot studies, over 100 items were condensed to the 22‐item version, of which 18 items were related to present self‐efficacy, and 4 related to future self‐efficacy. Each item is graded on an 11‐grade Likert scale ranging from 0 = *not at all certain* to 10 = *very certain*. A score is then calculated by averaging the items' scores for ‘present’ and ‘future’ self‐efficacy. The scale was reported to have high Cronbach's *α* (0.94), and factor analysis revealed the presence of two underlying factors, thought to represent ‘present’ and ‘future’ self‐efficacy. In 2020, a new, 18‐item version was published, where 4 items from the ‘present’ self‐efficacy sub‐scale were removed [8]. Items were removed due to feedback from patients and clinicians, which indicated that these items (jumping ashore, running after children, horseback riding, and moving around on a small boat) were either irrelevant or did not adequately reflect activities relevant to a majority of patients. In the new, 18‐item version (K‐SES_18_), content validity has not been explored; instead, structural validity, internal consistency, and construct validity have been assessed [8]. Construct and structural validity were assessed by hypothesis testing, and factor analysis by correlation with other scales (KOOS, Physical Activity Scale and ACL‐Return to Sport after Injury scale) [8]. Internal consistency was assessed with Cronbach's alpha for each subscale. Results show high alpha for the future subscale (0.81–0.91), and very high alpha (0.93‐0.96) for the present subscale, with risk for item redundancy, two factors with eigenvalues ≥ 1, and good construct validity as all seven predefined hypotheses were confirmed [8]. In this present study, the 18‐item version was assessed (Table 1).

**Table 1:** The 18 items of the K‐SES_18_ present and future.

K‐SES_18_ present
*A: daily activities*
How certain are you about:
1) Walking in the forest
2) Walking down stairs/down hill
3) Running to catch the bus
4) Working in the garden
5) Cleaning at home
*B: sport and leisure activities*
How certain are you about:
6) Cycling longer distance
7) Cross‐country skiing
8) Swimming
9) Dancing
10) Hiking in the mountains
*C: physical activities*
How certain are you about:
11) Squatting
12) Jumping sideways from one leg to the other
13) Hopping on the injured leg
14) Quickly changing direction

K‐SES_18_ future
*D: your knee function in the future*
How certain are you that:
1) Your knee will get well
2) You can return to the same physical activity level as before your injury
3) You will not have new knee injuries
4) Your knee will get better than before surgery (only if you have undergone surgery)

Abbreviation: K‐SES_18_, Knee Self‐Efficacy Scale (18‐item version).

### Patients

**Figure 1:**
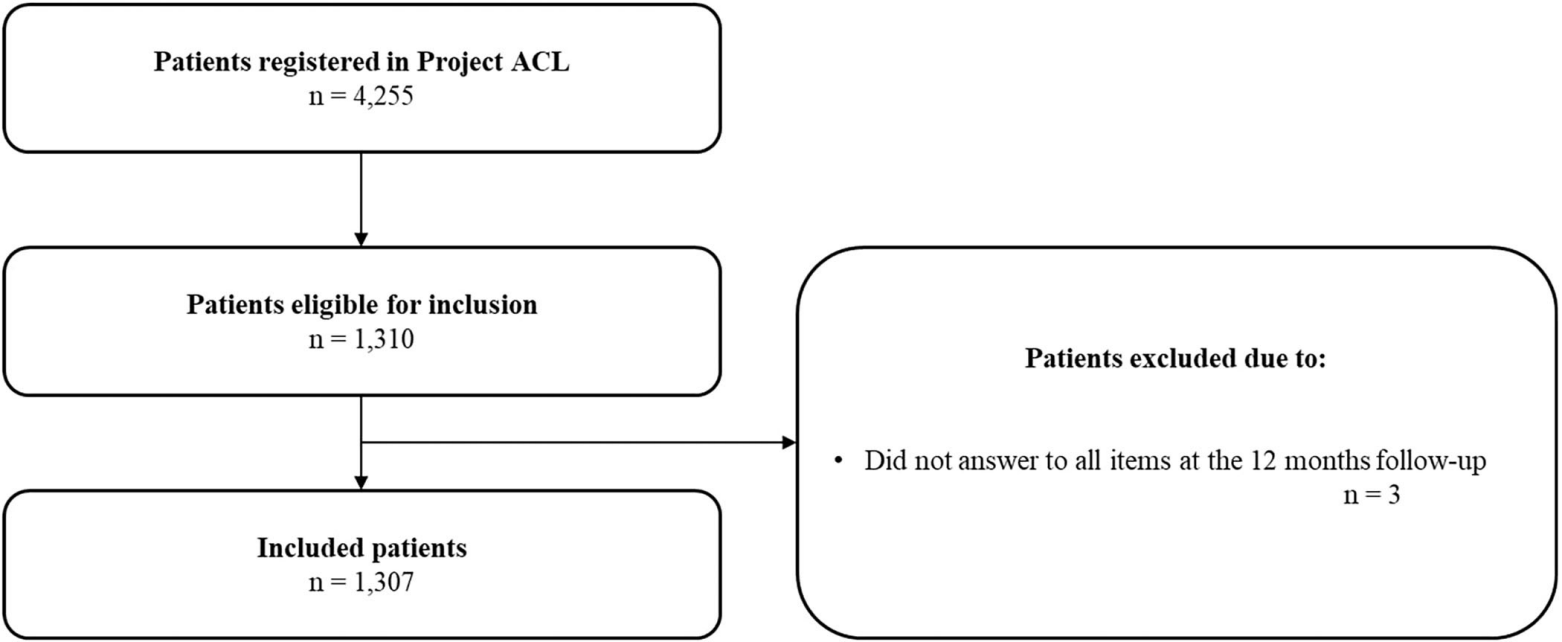
Flowchart of inclusion.

Patients registered in Project ACL, who had suffered one ACL injury treated with reconstruction, aged 15–50 at time of ACL reconstruction, and who completed the K‐SES_18_ at 12 months after ACL reconstruction were eligible for inclusion. Patients who did not answer all items of the K‐SES_18_ at the follow‐up were excluded (Figure 1).

Rehabilitation after ACL reconstruction typically lasts between 6 and 13 months before patients are discharged [24]. Given that 12 months falls within this timeframe and is commonly when patients complete their rehabilitation, this follow‐up point was selected for data extraction. Data was extracted from Project ACL on 29 February 2024.

Responses to the K‐SES_18_ were available from a total of 1307 patients in Project ACL. Table 2 presents patients demographics.

**Table 2:** Demographics of included patients.

	Total	Women	Men
Sex, *n*, (%)	1307	719 (55%)	588 (45%)
Age, years, mean (SD)	26.9 (9.1)	26.3 (9.3)	27.7 (8.5)
Height, cm, mean (SD)	174.2 (9.1)	168.3 (6.1)	181.5 (6.6)
Weight, kg, mean (SD)	72.9 (12.2)	66.1 (9.1)	81.1 (10.3)
Tegner pre‐injury level, *n* (%)			
1–5	213 (16.3%)	142 (19.8%)	71 (12%)
6	113 (8.6%)	73 (10.1%)	40 (6.8%)
7	224 (17.1%)	107 (14.9%)	117 (19.8%)
8	245 (18.7%)	150 (20.8%)	95 (16.1%)
9	352 (16.9%)	171 (23.7%)	181 (30.7%)
10	163 (12.4%)	77 (10.7%)	86 (14.6%)
Graft choice, *n* (%)			
Hamstring	955 (72.9%)	526 (73.1%)	429 (72.7%)
Patella	216 (16.5%)	113 (15.7%)	103 (17.5%)
Other	21 (1.7%)	12 (1.7%)	9 (1.5%)
Missing	118 (9%)	69 (9.6%)	49 (8.3%)

Abbreviations: cm, centimetres; kg, kilograms; *n*, number; SD, standard deviation.

### Analysis

Demographics for included patients were reported as number (*n*) and percentages (%) and mean and standard deviations (SDs). The RUMM2030plus software (RUMM Laboratory Pty Ltd) [4] was used to perform analysis on K‐SES_18_ item response data.

In RMT, persons, items and response category thresholds are located on a common log‐odds units (logit) scale according to their relative locations [5], and an individual's score on an item is determined by both the person's ability level and the difficulty level of the item response. This approach uses probability testing to account for item responses, to ensure a comprehensive consideration of a person's capability to answer each item [5]. We analyzed the K‐SES_18_ according to the unrestricted polytomous (partial credit) Rasch model [5]. The analyses focused on targeting (person‐item threshold distribution), item hierarchy, response category thresholds, overall and individual item and person fit, differential item functioning (DIF), unidimensionality and reliability. Bonferroni adjustments for multiple null hypothesis testing were applied, with the alpha level of significance set at *p* < 0.05 [9].

### Targeting and reliability

Targeting evaluates how well the difficulty range of the items aligns with the ability range of the individuals for the construct that is measured. Proper targeting guarantees that the instrument can capture the full range of the latent trait, which is important for content validity [32]. A mean person location of <±0.5 logits indicates that the instrument is well‐targeted, thus providing a measure that is neither too difficult nor too easy for the sample population [21].

Reliability of the scale is primarily assessed through the Person separation index (PSI), similar in interpretation to Cronbach's alpha. High PSI values signify that the scale can accurately differentiate respondents with different levels of the measured trait, which is an important facet to demonstrate internal consistency and overall reliability. Combining PSI with coefficient alpha offers a comprehensive evaluation of the scale's stability and ensures that it performs consistently across different groups [3]. In addition, since there were no missing responses in the data, coefficient alpha was also estimated.

### Response category thresholds

Response category thresholds are important to ensure that each category within a question is distinct and meaningful. Properly ordered thresholds, where respondents can reliably distinguish between adjacent categories, support content validity [32]. Disordered thresholds, conversely, indicate potential issues in how response options are perceived or understood by respondents, which could undermine the tool's effectiveness and accuracy. For example, if a scale includes response options from 0 to 10, but no respondents are most likely to select ‘5’ at any point along the self‐efficacy continuum, this suggests that the distinction between adjacent categories is unclear or unnecessary.

### Item hierarchy

The item hierarchy represents the ordered sequence of item difficulties, which should correspond with the expected theoretical structure of the construct being assessed. This sequence strengthens construct validity by showing that the tool can accurately distinguish between different levels of the trait in a logical and predictable manner [32]. The alignment of item difficulties with clinical and theoretical expectations verifies that the items adequately capture the construct. Patients with high scores on the K‐SES_18_ are expected to have high probabilities to endorse responses which represent high and positive knee‐related self‐efficacy, such as jumping sideways on one leg. Conversely, patients with low scores on the K‐SES_18_ are expected to have low probabilities to endorse such responses representing high levels of knee‐related self‐efficacy and may show higher agreement with items representing lower levels of knee‐related self‐efficacy.

### Overall, individual item and person fit: DIF

The overall model fit is evaluated through the mean standardized fit residuals for both items and persons, which should be near zero with a standard deviation close to one. The fit residuals indicate the deviation between observed and expected scores, while the chi‐square statistic tests these differences. A non‐significant chi‐square suggests a good model fit. Chi‐square values can inflate with large sample sizes. Thus, sample size adjustments that do not affect location or residuals can be applied [20]. Accordingly, a sensitivity analysis with an adjusted sample size of *n* = 500 was performed [17]. The sensitivity analysis was performed via random selection automatically performed by the software RUMM2030plus. Fit was also tested at the individual item and person level using the approximate chi‐square statistic as well as standardized fit residuals. Residuals between ±2.5 are generally considered acceptable [5].

High positive item fit residuals indicate multidimensionality within the scale. On the other hand, high negative fit residuals suggest local dependence between items. Local dependence between items means that item responses are influenced by other items, and not only driven by the underlying construct [22]. Thus, items measure more than just the intended latent trait [22]. Local dependency may be due to multidimensionality or that the response to one item affects response(s) to other item(s). Local dependency can introduce bias into the measurement process and artificially inflate reliability indices. To investigate local dependency in this study, item residual correlations were analyzed. A critical value (CV) of 0.1, derived from the 99th percentile, was used for robust evaluation. The CV was determined using a web‐based application developed based on the methodology proposed by Christensen et al. [11] (http://publicifsv.sund.ku.dk/~kach/Q3/critical_values_Yens_Q3.html).

DIF was also assessed to examine potential biases in item performance across subgroups. DIF occurs when items are interpreted or answered differently by individuals with comparable levels of the measured construct but belonging to distinct demographic groups [12]. A DIF factor should not be a factor that is comprised within the construct the scale aims to measure [18]. To ensure that the scale measures the intended construct consistently across populations, DIF was analyzed for sex (men and women) and age groups. Age was categorized into two groups: younger (15–25 years) and older (26–50 years), based on the median age of the sample.

### Unidimensionality

Unidimensionality ensures that a set of items measures a single underlying construct, which is important for construct validity, and indicates that the total score represents a single dimension rather than multiple unrelated traits. To verify unidimensionality, this study used PCA of residuals to create two item sets, then estimated separate person measures for each set of items. The measures were compared using *t* tests, and if ≤5% of tests were significant, or the 95% confidence interval (CI) included 5%, unidimensionality was inferred [16]. In this study, the binomial 95% CI was calculated according to Agresti and Coull [1]. A sub‐analysis was performed grouping items according to the domain of belonging when the scale was developed. The change in the reliability estimate (compared to the initial item‐level analysis) indicates the degree of dependency, and the indices *c r* and *A* can be used to address dimensionality. *A* describes the non‐error variance common to all subtests, *c* characterizes the variance that is unique to the subtests (relative to the common variance = 1) and *r* is the latent correlation between the subtests.

## RESULTS

### K‐SES present

#### Targeting and reliability

**Figure 2:**
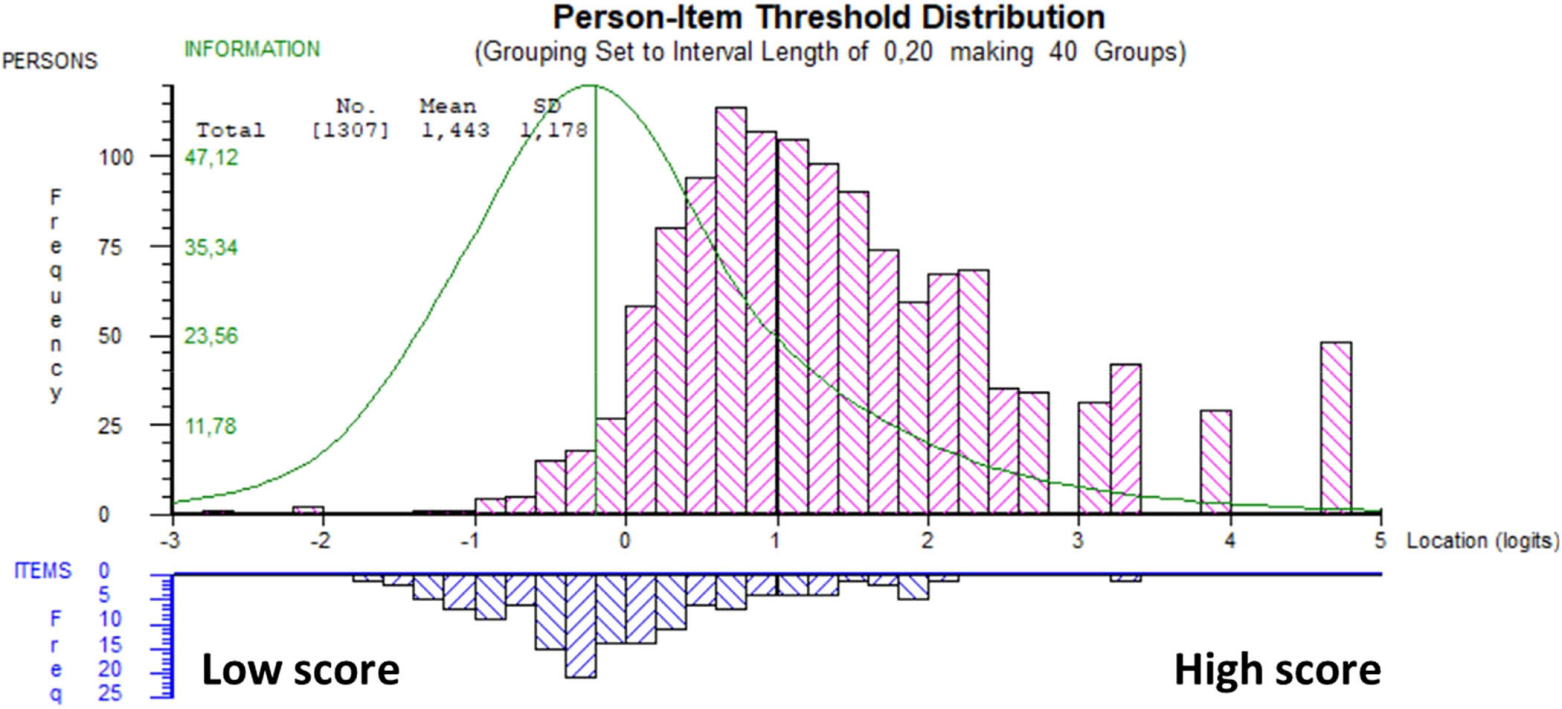
Person‐item threshold distribution for the K‐SES_18_ present subscale, distribution of people (upper panel) and response category thresholds (lower panel) on the common logit metric (*x*‐axis; positive values = more positive knee self‐efficacy. K‐SES, Knee Self‐Efficacy Scale.

For the K‐SES_18_ present, the mean (SD) person location was 1.440 (1.177). This can result in a ceiling effect where differences among higher ability individuals are not effectively measured (Figure 2). Estimations of reliability showed a PSI value of 0.904 and *α* of 0.939, indicating good reliability of the K‐SES_18_ present.

#### Response category thresholds

**Figure 3:**
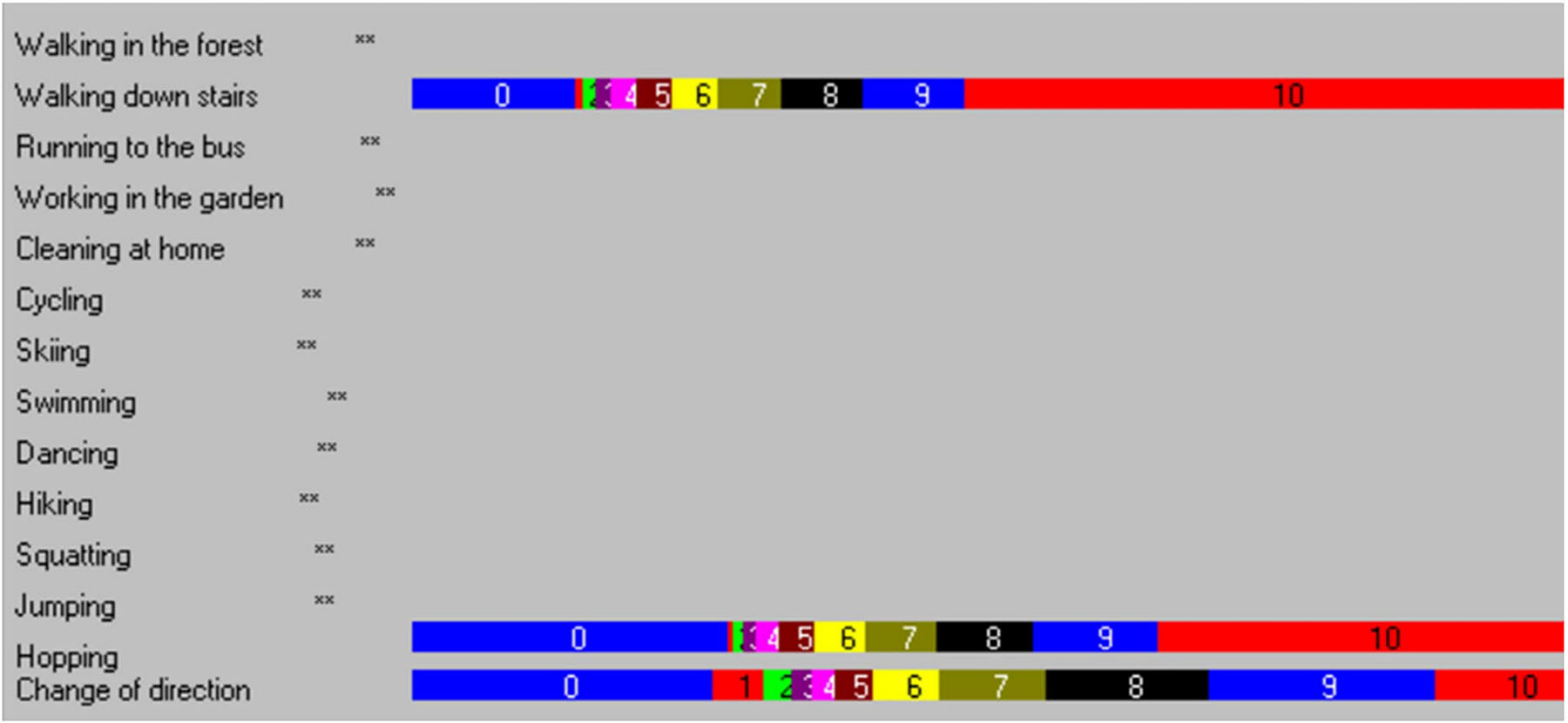
Threshold map; xx = disordered thresholds.

Items 2 (walking down stairs), 13 (hopping on the injured leg) and 14 (quickly changing direction) did not show disordered thresholds. All other 11 items of the K‐SES_18_ present showed disordered thresholds (Figure 3).

#### Item hierarchy

Sorting items by location order indicated that ‘cleaning at home’ (Item 5), with a location of −0.967 logits, is the easiest task. This implies that most patients in our sample find this activity to be the least challenging, and it requires the lowest level of knee self‐efficacy. On the other hand, ‘cross‐country skiing’ (Item 7) with a location of 0.819 logits is the most difficult task, indicating that it requires the highest level of knee self‐efficacy for a person to perform or agree with.

#### Overall and individual item and person fit: DIF

Overall fit assessment showed misfit to the Rasch model: total‐item *χ* ^2^ = 759.8, *p* < 0.0001, item mean (SD) fit residual = −0.27 (5.8) and person mean (SD) fit residual = −0.31 (1.1). A mean fit residual close to 0 suggests that overall, items fit the model reasonably well. However, the large standard deviation (5.8) indicates substantial variability in how individual items fit the model. The chi‐square for the sample adjusted to *n* = 500 was 301.6, *p* < 0.0001, which still indicated misfit to the model.

**Figure 4:**
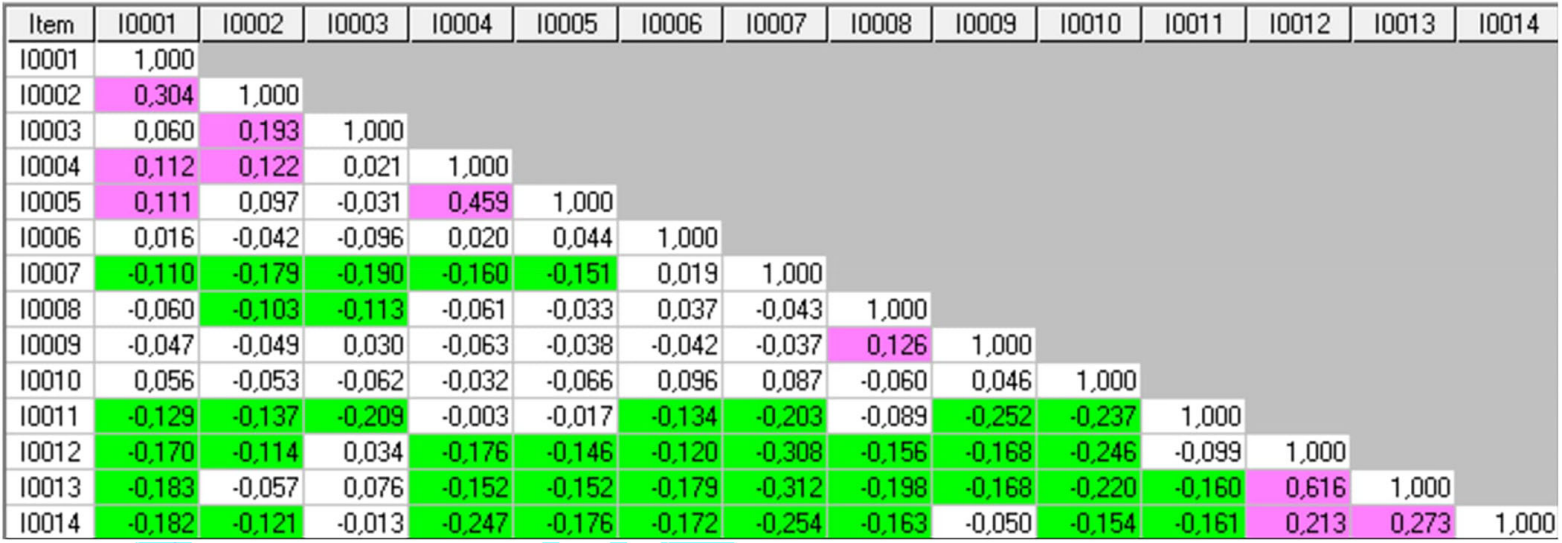
Inter‐items correlations matrix in K‐SES_18_ present. Green highlight: negative values above critical value; pink highlight: positive values above critical value. K‐SES, Knee Self‐Efficacy Scale.

Table 3 shows the fit residuals for each item. Items 4, 8, 10 and 14 did not signal misfit. After sample size adjustment, Items 3 (running to catch the bus), 9 (dancing), 11 (squatting) and 7 (cross‐country skiing) still indicated misfit, with Items 11 (squatting) and 7 (cross‐country skiing) strongly signalling multidimensionality. The CV was 0.1. Many different inter‐item correlations were found (Figure 4).

**Table 3:** Item hierarchy and fit residuals for each item of the K‐SES present.

Item number	Description	Location	Fit residual	Chi‐square	*p*	*p* adjusted sample size (*n* = 500)
5	Cleaning at home	−0.967 (0.036)	−2.722	34.745	<0.001	0.129
1	Walking in the forest	−0.693 (0.029)	−3.702	35.826	<0.001	0.114
2	Walking down the stairs	−0.519 (0.027)	−3.222	35.986	<0.001	0.112
6	Cycling longer distances	−0.507 (0.027)	−2.568	33.117	<0.001	0.155
4	Working in the garden	−0.403 (0.027)	−2.029	31.048	<0.001	0.195
3	Running to catch the bus	−0.120 (0.023)	−5.139	56.876	<0.001	0.007
8	Swimming	−0.078 (0.022)	1.545	27.772	0.001	0.273
12	Jumping sideways	0.258 (0.020)	−3.923	36.766	<0.001	0.102
13	Hopping on the injured leg	0.275 (0.020)	−2.795	20.257	0.01	0.529
9	Dancing	0.278 (0.021)	−4.180	45.980	<0.001	0.032
10	Hiking in the mountains	0.286 (0.020)	−1.329	24.211	0.003	0.382
11	Squatting	0.648 (0.018)	13.771	219.712	<0.001	<0.001
14	Quickly changing direction	0.723 (0.020)	0.725	26.865	<0.001	0.299
7	Cross‐country skiing	0.819 (0.017)	11.682	130.668	<0.001	<0.001

*Note*: Fit residuals should be between ±2.5. Items are sorted by location. *n* = number; *p* value adj = *p* value adjusted for size, where size was reduced to 500 individuals.

Abbreviation: K‐SES, Knee Self‐Efficacy Scale.

High positive correlations (Figure 4) were found between:

‐ Items 1 (walking in the forest) and 2 (walking down the stairs) (0.304),
‐ Items 4 (working in the garden) and 5 (cleaning at home) (0.459),
‐ Items 12 (jumping sideways) and 14 (quickly changing direction) (0.213),
‐ Items 13 (hopping on the injured leg) and 14 (quickly changing direction) (0.273), and
‐ Items 12 (jumping sideways) and 13 (hopping on the injured leg) (0.616).

High positive correlations can indicate local dependency.

No DIF by sex or age was found.

#### Unidimensionality

PCA of fit residuals identified three major factors on which items loaded (eigenvalues 2.427, 1.187, 1.502). By the third factor, 41.04% of the variance was explained, which suggests that there are several meaningful dimensions assessed in the K‐SES_18_ present. According to loading, Items 1 (walking in the forest), 2 (walking down the stairs), 4 (working in the garden), 5 (cleaning at home) and 7 (cross‐country skiing) were grouped into one group. Items 12 (jumping sideways from one leg to the other), 13 (hopping on the injured leg) and 14 (quickly changing direction) were grouped into another group. This showed significantly different locations for 9.5% (Agresti–Coull 95% CI = 8.8–11) of individuals, signalling multidimensionality.

### Subgroup analysis

To account for local dependency in K‐SES_18_ present, items were grouped according to domain of creation. Items 1–5 were grouped in a ‘daily activity’ group, Items 6–10 in a ‘leisure time’ group and Items 11–14 in a ‘physical activity’ group. Reliability was unchanged.

Fit residuals were comprised between ±2.5: daily activity −1.469 (SE: 0.009); leisure time: −0.119 (SE: 0.007) and physical activity 0.212 (SE: 0.007). However, fit residual correlations were still above CVs (Table 4).

**Table 4:** Inter‐item correlation matrix when items were grouped according to domain of creation.

Item group	Daily activity	Leisure time	Physical activity
Daily activity	1.000		
Leisure time	−0.266	1.000	
Physical activity	−0.350	−0.760	1.000

No significant differences in locations for individuals were found between item groups for ‘daily activity’ and ‘leisure time’ (0.54%), ‘daily activity’ and ‘physical activity’ (1.84%) and leisure time and physical activity (3.44%), which signals unidimensionality. This is in accordance with the *c* (0.56), *r* (0.75) and *A* (0.91) indices, where a low value for *c* and high values for *r* and *A* suggest unidimensionality.

In summary, after accounting for local dependency, presumptions for unidimensionality were respected; however, there was still a high correlation between item groups, which indicates that local dependency was not entirely absorbed.

### K‐SES future

#### Targeting and reliability

**Figure 5:**
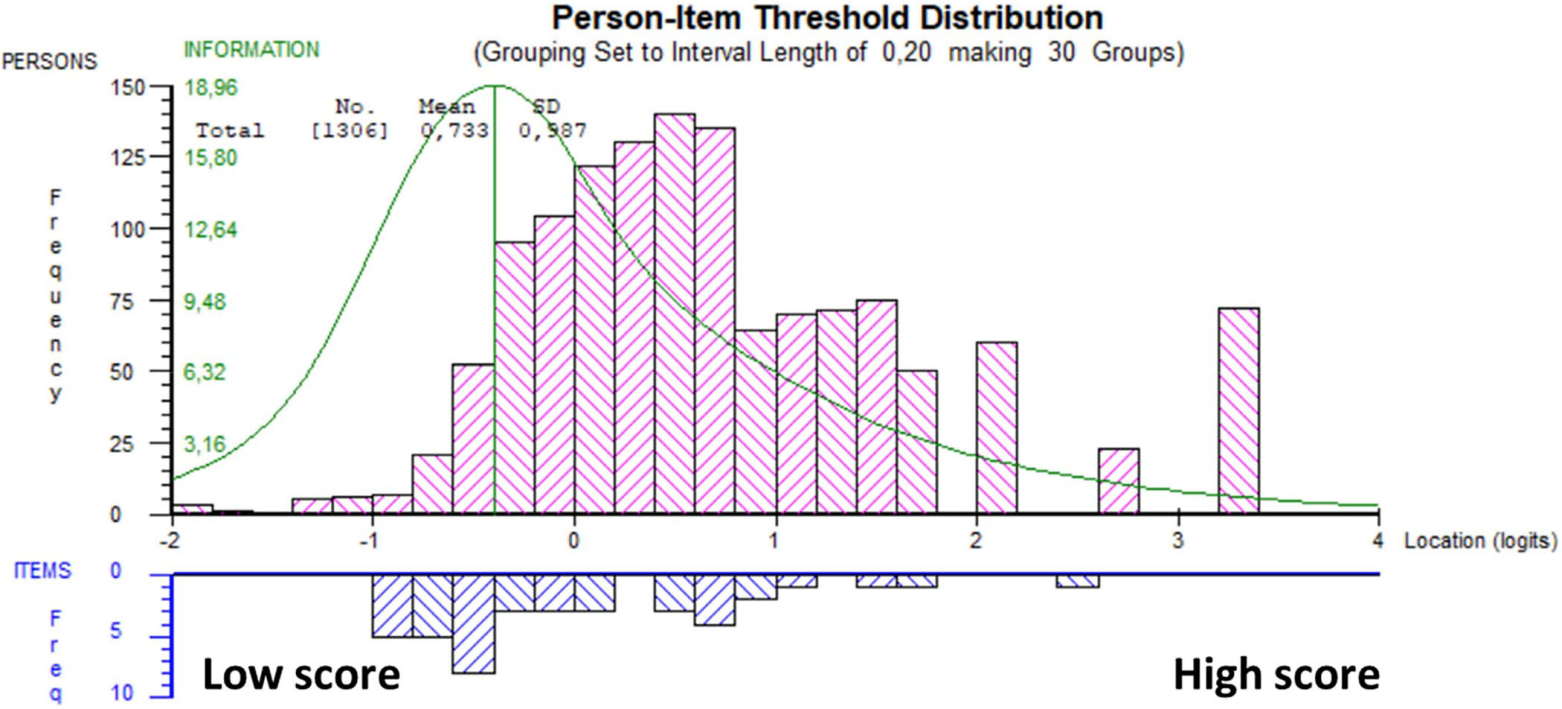
Person‐item threshold distribution for the K‐SES_18_ future subscale, distribution of people (upper panel) and response category thresholds (lower panel) on the common logit metric (*x*‐axis; positive values = more positive knee self‐efficacy. K‐SES, Knee Self‐Efficacy Scale.

For the K‐SES_18_ future, the mean (SD) person location was 0.733 (0.987), which suggests that, on average, the abilities of the patients are moderately above the average difficulty of the test items (Figure 5). Estimations of reliability showed a PSI value of 0.750 and *α* of 0.783, indicating good reliability of the K‐SES_18_ future.

#### Response category thresholds

All four items in the K‐SES_18_ future showed disordered thresholds.

#### Item hierarchy

The item hierarchy for the K‐SES_18_ future subscale (Table 5) showed that ‘Your knee will get well’ was the easiest item (lowest location value), while ‘Your knee will be better than before surgery’ was the most difficult item (highest location value). This suggests that patients were more confident in their knee recovering to a ‘well’ level but less certain about achieving an outcome superior to their pre‐injury state. Item fit analysis revealed that ‘Your knee will get well’ and ‘You will not have new knee injuries’ exhibited misfit to the Rasch model, indicating potential issues with how these items function within the scale.

**Table 5:** Item hierarchy and fit residuals for each item of the K‐SES future.

Item number	Description	Location	Fit residual	Chi‐square	*p*	*p* adjusted sample size (*n* = 500)
1	Your knee will get well	−0.409 (0.022)	−3.715	77.044	<0.001	<0.001
2	Return to physical activity	−0.255 (0.019)	−2.333	55.767	<0.001	0.007
3	Not have new knee injuries	0.229 (0.015)	3.359	19.448	0.02	0.792
4	Better than before surgery	0.435 (0.018)	2.513	13.436	0.14	0.544

*Note*: Fit residuals should be between ±2.5. Items are sorted by location. *n* = number; *p* value adj = *p* value adjusted for size, where size was reduced to 500 individuals.

Abbreviation: K‐SES, Knee Self‐Efficacy Scale.

#### Overall and individual item and person fit: DIF

Overall fit assessment of the K‐SES_18_ future showed some misfit to the Rasch model: total‐item *χ*2 = 165.6, *p* < 0.0001, item mean (SD) fit residual = −0.04 (3.5) and person mean (SD) fit residual = −0.44 (1.1). The chi‐square for the adjusted sample was 67.3, *p* = 0.001. The large SD in items' mean fit residuals indicate that some items do not fit the model well.

Table 5 displays fit residuals for each item. Items 1 (your knee will get well) and 3 (you will not have new knee injuries) had fit residuals above ±2.5, which indicates misfit. After sample size adjustment, Item 1 still signalled misfit, while Item 3 was no longer significant.

**Figure 6:**
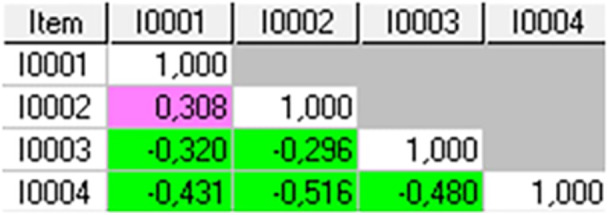
Inter‐item correlations matrix for items in K‐SES future. Green highlight: negative values above critical value; pink highlight: positive values above critical value. K‐SES, Knee Self‐Efficacy Scale.

All items displayed correlation coefficients above CV (0.1) (Figure 6). Most correlations were negative, which indicates multidimensionality.

No DIF by sex or age was found.

#### Unidimensionality

PCA of fit residuals identified at least two major factors on which items loaded (eigenvalues 1.846, 1.432). Two factors explained 82% of the variance, and the third (eigenvalue 0.690) added 17.2%. According to loading, Items 1 and 2 were grouped into one group, and Items 3 and 4 into a second group. This showed significantly different locations for 3.45% (Agresti–Coull 95% CI = 2–4) of individuals, which implies that unidimensionality can be present.

## DISCUSSION

This is the first comprehensive analysis of the K‐SES_18_ based on RMT [10, 38]. The analysis shows that both the present and future subscale have good reliability, however, the analysis also unveils significant challenges related to targeting, dimensionality, and item response behaviour, which bear implications for clinical application and possible scale refinement.

### K‐SES_18_ present

The K‐SES_18_ present shows good reliability and has been reported to predict return to activity and self‐reported function in activity after ACL reconstruction [36]. However, the K‐SES_18_ present demonstrates notable challenges with targeting and response thresholds. The mean person location of 1.440 suggests a potential ceiling effect, which risks underestimations of improvement for patients with higher self‐efficacy and possibly results in a ‘washout’ effect at higher scale levels. While high self‐efficacy is generally desirable, it is important for the scale to distinguish between appropriate levels of confidence and potentially maladaptive overconfidence, which might increase the risk of injury [28]. This ceiling effect limits the scale's sensitivity in tracking improvement in patients over time. As a result, clinicians should be cautious when interpreting high K‐SES_18_ present scores, as high scores may not reflect continued functional progress but rather a measurement limitation. Conversely, the absence of floor effects highlights the scale's strength in identifying patients with low self‐efficacy who may benefit most from targeted interventions. Additionally, disordered thresholds in 11 of the 14 items suggest that respondents may struggle to distinguish between adjacent response categories, which limits the scale's sensitivity to detect minor yet clinically significant changes in self‐efficacy. These challenges are crucial markers of limited content validity. One important step in the development of content validity on a scale is the engagement of patients, that is, the ultimate stakeholders in a scale development, during the scale's development [29]. In the original paper from 2005, some items were reported to be generated by ‘discussion between health care professionals and patients’ [35]. However, how many patients, how, and at what stage they participated was not reported, and consequently, the K‐SES was reported to have limited evidence due to poor methodological quality [15].

Item hierarchy analysis further reveals that daily activities (Items 1–5), such as cleaning at home, are perceived as less challenging, which aligns with clinical expectations. However, item hierarchy analysis placed items 11 (squatting) and 7 (cross‐country skiing) in the last position, which indicates that patients struggle to have a high level of self‐efficacy in these activities. Notably, ‘squatting’ was more difficult to achieve than ‘hopping on the injured leg’. Furthermore, Items 7 and 11 exhibited a strong misfit to the Rasch model, suggesting they may not be functioning as intended within the context of the scale. To improve scale validity and applicability, the re‐evaluation and potential removal of these misfitting items could be advised.

Four out of fourteen items did not signal misfit, and after sample size adjustments, Items 3 (running to catch the bus), 9 (dancing), 11 (squatting) and 7 (cross‐country skiing) still indicated misfit, with Items 11 (squatting) and 7 (cross‐country skiing) strongly signalling multidimensionality. One possible reason for the misfit of several items could be the non‐specificity of the level of activity. The word ‘dancing’ can imply a span of activities ranging from Valzer to breakdance. Similarly, ‘squatting’ can encompass a range of activities from sitting on a chair to powerlifting. It is reasonable that in the context of knee‐related self‐efficacy, patients might struggle to answer too unspecified physical activities, such as ‘dancing’, which in such a case will produce answers of limited validity. Nevertheless, clinicians could use answers to each specific question to introduce a discussion with each specific patient about which level and how the patient resonated upon providing an answer.

The PCA of the K‐SES_18_ present indicated a complex structure with three major factors explaining 41.04% of the variance. Given that three distinct factors emerged in this study, these findings suggest that the K‐SES_18_ present subscale may function more accurately as separate subscales rather than a single total score. This would align with the multidimensional nature of self‐efficacy in ACL rehabilitation, where different activities may require distinct psychological and functional confidence. While the K‐SES_18_ has traditionally been used as a total score, our findings indicate that this approach may not be psychometrically sound, as the presence of multiple factors challenges the assumption of unidimensionality. This multidimensionality within the scale suggests that knee‐related self‐efficacy as measured by the K‐SES_18_ present encompasses several distinct dimensions that might reflect different aspects of how certain a patient is to perform various activities after ACL reconstruction. Items such as ‘walking in the forest’, ‘walking down stairs’, ‘working in the garden’ and ‘cleaning at home,’ loaded on one factor. These activities generally involve basic mobility and daily functioning, which may represent a foundational level of knee self‐efficacy related to routine and less physically demanding tasks. In contrast, the second factor, including ‘jumping sideways’, ‘hopping on the injured leg’, and ‘quickly changing direction’, represents more dynamic and physically demanding activities that likely require higher levels of certainty that the patient has enough physical function. Accordingly, the calculation of a single score for the K‐SES_18_ present is biased, and patients are not adequately challenged by all items, which results in less precise measurements above a certain level of knee‐related self‐efficacy. Given these considerations, the construct validity of the K‐SES_18_ present can be defined as limited, even though no DIF for age or sex was found.

For the construct validity of the K‐SES_18_ present, one important distinction to be made is between self‐efficacy and confidence. Self‐efficacy is described as an individual's confidence in their ability to perform actions required to achieve specific outcomes [6]. In the context of rehabilitation following ACL reconstruction, self‐efficacy plays a critical role, as it encompasses the patient's ability to execute physical activities, such as jumping or running. A deficiency in self‐efficacy can negatively influence the results of treatment [27]. Confidence, on the other hand, refers to a retrospective meta‐cognitive judgment [14], which is a reflective assessment or evaluation of one's own thought processes, knowledge, and abilities for a given situation. Therefore, confidence is derived from looking back at past experiences and assessing one's own ability to have dealt with given situations successfully. Confidence, in the context of sports injury rehabilitation, relates to a patient's trust or belief in the overall athletic abilities, including the ability to perform well in the sport despite the injury. Confidence extends beyond the specific tasks associated with rehabilitation, for which self‐efficacy is important, to encompass a broader sense of competence [14]. There is a possibility that since patients respond to the K‐SES in a safe environment, the scale reflects more patients' confidence than their self‐efficacy, which could partly explain the misfit to the model.

The subgroup analysis in this study attempted to address local dependency by grouping items into domains: daily activity, leisure time and physical activity. While this approach partially respected unidimensionality within groups, negative correlations between group fit residuals persisted, highlighting underlying contrasts. This suggests that while each group might measure a facet of knee‐related self‐efficacy, they represent distinct dimensions that prevent aggregation into a single score.

### K‐SES future

The K‐SES_18_ future exhibits good reliability (PSI = 0.750, *α* = 0.783), yet its mean person location of 0.733 implies the scale could be slightly easy for the average patient, which could impact the clinical usefulness to identify patients with different levels of future knee self‐efficacy. As the K‐SES_18_ present scale, disordered thresholds and significant misfit in items such as ‘your knee will get well’ and ‘you will not have new knee injuries’ challenge the scale's item functioning. Additionally, a PCA revealed at least two dimensions, complicating the scale's presumed unidimensionality. Despite these dimensions, less than 5% difference in PCA *t* tests might allow the scale to function as unidimensional for certain analyses, though the negative item correlations suggest otherwise.

### Clinical implications

For the K‐SES_18_ present, the identification of multiple underlying dimensions and the high reliability of the questionnaire suggest that it can be a valuable tool for assessing specific aspects of knee self‐efficacy in clinical practice. However, clinicians should be cautious about using total scores to make clinical decisions. Instead, to interpret the three different sub‐domains (‘daily activity’, ‘leisure time’ and ‘physical activity’) separately might provide more nuanced insights into the areas where patients feel most and least self‐efficacy, which could facilitate more targeted interventions. Moreover, understanding the distinct dimensions of self‐efficacy can guide clinicians to provide psychological support where it is most needed to enhance overall treatment outcomes.

For the K‐SES_18_ future, the misfit and disordered thresholds suggest that clinicians should use the KSES_18_ future scores cautiously.

Individual item analysis may be more informative than a composite score until the scale's issues are addressed, which include the revision of scoring categories, the re‐evaluation of misfitting items, multidimensionality and targeting issues. Clinicians should consider to assess responses to individual items on the K‐SES_18_ as a foundation for patient‐centred discussions. Each item reflects a specific aspect of knee‐related self‐efficacy, which might offer valuable insights into areas where patients could feel confident and areas where patients may experience uncertainty or hesitation. To assess item‐specific responses, clinicians can identify tasks or activities where self‐efficacy could be strengthened, such as challenging physical activities, or potentially moderated, such as overly optimistic beliefs that may predispose the patient to risky behaviours.

### Limitations

One limitation of the Rasch analysis is the assumption of unidimensionality: the model assumes that the data analyzed measures a single underlying trait or dimension, which can be restrictive because many psychological constructs are inherently multidimensional. We included over 1000 patients in this study, which is sufficiently large for a Rasch analysis [25]. The study population was heterogeneous in terms of sex and age, the two variables considered in the DIF analysis. However, we did not account for several potential confounding factors that could influence responses to the K‐SES_18_, including RTS, type of sport, pre‐injury activity level and concomitant injuries. These factors could have influenced self‐efficacy scores. Athletes who participated in high‐level competitive sports before ACL injury may have developed greater confidence in their ability to recover, leading to higher K‐SES_18_ scores. Conversely, patients with concomitant injuries or patients engaged in less physically demanding activities before ACL injury might have reported lower self‐efficacy due to greater uncertainty about recovery. Future studies should explore how factors such as RTS, type of sport, pre‐injury activity level, and concomitant injuries interact with self‐efficacy to refine the interpretation of K‐SES_18_ scores in different patient subgroups. Additionally, the follow‐up period was set at 12 months post‐ACL reconstruction—a typical timeframe for returning to sport—though outcomes might vary at different follow‐up intervals, such as 8 or 24 months post‐surgery. Despite these limitations, we believe the sample adequately represents the diversity of patients undergoing ACL reconstruction. Our findings are robust but should be generalized with caution.

### Conclusion

The main finding of this study is that the K‐SES_18_ according to RMT demonstrates strong reliability but has limitations in terms of measurement properties that possibly limit its clinical utility. The present and future subscales exhibit ceiling effects, disordered thresholds, and item misfit. Therefore, the K‐SES_18_ might not be able to capture the full range of patients' knee self‐efficacy after ACL reconstruction. These findings indicate that a scale refinement to enhance targeting, response sensitivity and dimensionality is needed. Clinicians are therefore advised to interpret the subscale scores cautiously, acknowledging the multidimensional nature of the constructs measured.

## AUTHOR CONTRIBUTIONS

**Ramana Piussi**: Conceptualization; methodology; formal analysis; investigation; writing—original draft preparation; writing—review and editing; funding acquisition. **Eric Hamrin Senorski**: Conceptualization; methodology; writing—review and editing; resources; supervision. **Roland Thomeé**: Writing—review and editing; resources. **Kristian Samuelsson**: Writing—review and editing; resources; supervision.

## CONFLICT OF INTEREST STATEMENT

Author Kristian Samuelsson discloses he is a board member of Getinge AB (publ). The remaining authors declare no conflicts of interest.

## ETHICS STATEMENT

Ethical approval was obtained from the Regional Ethical Review Board in Gothenburg (265‐13, T023‐17) and the Swedish Ethical Review Authority (2020‐02501).

## Data Availability

The data that support the findings of this study are available on request from the corresponding author. The data are not publicly available due to privacy or ethical restrictions.
